# Utilizing genetic code expansion to modify N-TIMP2 specificity towards MMP-2, MMP-9, and MMP-14

**DOI:** 10.21203/rs.3.rs-2446107/v1

**Published:** 2023-01-16

**Authors:** Hezi Hayun, Matt Coban, Ashok Kumar Bhagat, Eden Ozer, Lital Alfonta, Thomas R. Caulfield, Evette S. Radisky, Niv Papo

**Affiliations:** Ben-Gurion University of the Negev; Mayo Clinic Comprehensive Cancer Center; Ben-Gurion University of the Negev; Ben-Gurion University of the Negev; Ben-Gurion University of the Negev; Mayo Clinic College of Medicine; Mayo Clinic Comprehensive Cancer Center; Ben-Gurion University of the Negev

**Keywords:** Binding specificity, matrix metalloproteinase, protease inhibitor, protein engineering, protein-protein interactions (PPIs), proteolysis, molecular modeling, non-canonical amino acids

## Abstract

Matrix metalloproteinases (MMPs) regulate the degradation of extracellular matrix (ECM) components in biological processes. MMP activity is controlled by natural tissue inhibitors of metalloproteinases (TIMPs) that non-selectively inhibit the function of multiple MMPs via interaction with the MMPs’ Zn^2+^-containing catalytic pocket. Recent studies suggest that TIMPs engineered to confer MMP specificity could be exploited for therapeutic purposes, but obtaining specific TIMP-2 inhibitors has proved to be challenging. Here, in an effort to improve MMP specificity, we incorporated the metal-binding non-canonical amino acids (NCAAs), 3,4-dihydroxyphenylalanine (L-DOPA) and (8-hydroxyquinolin-3-yl)alanine (HqAla), into the MMP-inhibitory N-terminal domain of TIMP2 (N-TIMP2) at selected positions that interact with the catalytic Zn^2+^ ion (S2, S69, A70, L100) or with a structural Ca^2+^ ion (Y36). Evaluation of the inhibitory potency of the NCAA-containing variants towards MMP-2, MMP-9 and MMP-14 *in vitro* revealed that most showed a significant loss of inhibitory activity towards MMP-14, but not towards MMP-2 and MMP-9, resulting in increased specificity towards the latter proteases. Substitutions at S69 conferred the best improvement in selectivity for both L-DOPA and HqAla variants. Molecular modeling revealed how MMP-2 and MMP-9 are better able to accommodate the bulky NCAA substituents at the intermolecular interface with N-TIMP2. The models also showed that, rather than coordinating to Zn^2+^, the NCAA side chains formed stabilizing polar interactions at the intermolecular interface with MMP-2 and MMP-9. The findings illustrate how incorporation of NCAAs can be used to probe and exploit differential tolerance for substitution within closely related protein-protein complexes to achieve improved specificity.

## Introduction

Human matrix metalloproteinases (MMPs) comprise a family of 28 known zinc-dependent catalytic enzymes that play major roles in the degradation of extracellular matrix (ECM) components. The role of MMPs in ECM remodeling renders them significant players in biological processes as diverse as angiogenesis, tissue hemostasis, wound healing and embryogenesis^1^. MMPs are thus attractive as therapeutic targets, particularly since imbalances in MMP activity or expression can promote pathological conditions, such as arthritis, cardiovascular diseases, and cancer progression, invasion and metastasis^1–4^. For example, among the MMP family, MMP-2, MMP-9 and MMP-14 are expressed in 70–100% of invasive breast tumors. The differential expression of these MMPs as markers for different diseases, including different cancers, and the ability of this expression to change over time highlight the importance of developing tailored therapeutic strategies for their selective inhibition^5^. Although belonging to different subgroups of MMPs, the gelatinases, MMP-2 and MMP-9, and the membrane-type MMP, MMP-14, exhibit high similarity in their sequences and structures. This similarity is paradoxically both an advantage and a disadvantage; the former because their X-ray structures have been solved and are available for bioinformatic analysis of their interactions with each other and with their inhibitors and substrates^6^, and the latter because the design of specific inhibitors becomes a very challenging task.

One of the structural characteristics that is common to all MMP family members is the catalytic domain, which has a conserved zinc-binding motif, HEXGHXXGXXH, a catalytic Zn^2+^ ion, a structural Zn^2+^ ion, and two (or three) Ca^2+^ structural ions, all of which contribute to its stabilization. The catalytic Zn^2+^ ion is coordinated to three His residues of the conserved motif and to one water molecule. When the catalytic domain binds to a substrate, the coordinated water molecule becomes polarized between the conserved catalytic glutamate base in the zinc-binding motif and the catalytic Zn^2+^ Lewis acid to facilitate a nucleophilic attack on a peptide bond, resulting in substrate hydrolysis^7^. Substrate specificity is determined by the size and shape of the six contact sites surrounding catalytic Zn^2+^ ion^1,8–10^. Of these, the S1 specificity pocket differs most in size, shape and amino acid content among the different MMPs, and substrates (or inhibitors) with complementary properties at this site may exhibit higher affinity and selectivity for particular MMPs over others.

The interaction of MMPs with the four tissue inhibitors of metalloproteinases (TIMP1–TIMP4) is an important mechanism by which MMP activity is regulated in vivo^11,12^, with the major TIMP-MMP interaction taking place through the binding of the N-terminus of the TIMP (Cys1–Pro5) to the S1, S1 , S2 , S3 , and S4 pockets of the MMP^13^. Specifically, the Cys1 residue (which is bound to Cys72 via a disulfide bond) interacts with the catalytic Zn^2+^ ion of the MMP via its N-terminal α-amino and carbonyl groups, displacing the water molecule and thus serving as a fourth zinc ligand. Cys1 also interacts with the catalytic glutamic acid residue of the zinc-binding motif via a hydrogen bond. The second residue of the TIMP, being either threonine or serine^14^, can also form a hydrogen bond with the catalytic glutamic acid, as may be seen in some, but not necessarily all, crystal structures^15^. Several studies have thus set out to manipulate this MMP-TIMP interaction as a means of improving its specificity, for example, by replacing Ser2 with Glu or Asp20 orSer68 with Arg21^16,17^. In particular, considerable effort has been devoted to developing high-affinity inhibitors with good specificity for particular MMPs for therapeutic applications. However, the potential of most synthetic MMP inhibitors^9,18–20^ that were designed to chelate the catalytic Zn^2+^ ion has not been realized: these synthetic compounds exhibit good inhibition activity, but their selectivity is limited and they are not suitable for clinical use due to poor solubility^21^, poor pharmacokinetics, low bioavailability and severe adverse effects^22,23^. In contrast, TIMP2 and its isolated N-terminal domain, N-TIMP2, have shown promising inhibition potency towards the MMP family in that they exhibit high affinity toward various MMPs (10^− 12^–10^− 9^ M), and – being native human proteins – they are likely to be non-toxic and non-immunogenic. However, although N-TIMP2 shows high affinity to MMPs, it lacks specificity for particular MMPs. We therefore sought to improve the specificity by using genetic code expansion, in which non-canonical amino acids (NCAAs) are site-specifically incorporated into target proteins. The rationale for this approach is based on previous studies in which NCAAs were used to improve the selectivity of different peptides and proteins. For example, 4-tert-butyl and 4-aminomethyl derivatives of phenylalanine were shown to have 20- to 30-fold higher affinity than phenylalanine for their synthetic receptor (Q7), respectively^24^. Previous studies have also shown that the incorporation of NCAAs into proteins and small peptides can be used to improve and to tune their metal binding affinities^25^, the importance of which derives from the crucial roles of metal ions in many biological processes, such as apoptosis, oxidative stress, and immune defense.

Here, we sought to incorporate NCAAs into N-TIMP2 so as to differentially modulate inhibition of different MMPs in a manner that would enhance specificity. We leveraged the NCAAs to probe the potential of bulky polar residues with metal-binding capability either to differentially enhance MMP binding or to be differentially tolerated by different MMPs. The two metal-binding NCAAs that we judged to be suitable for this study were 3,4-dihydroxyphenylalanine, also known as L-DOPA or hydroxytyrosine, which has a catechol side chain, and (8-hydroxyquinolin-3-yl)alanine (HqAla), which has a derivative of the 8-hydroxyquinoline chelating moiety in its side chain. Our choice of these two NCAAs was based on previous studies showing that catechol-based compounds^26–28^ and 8-hydroxyquinoline derivatives^29,30^ exhibited promising potential as inhibitors of MMP-2, MMP-9 and MMP-14, with IC_50_ values in the low and even the sub-micromolar range, and that they displayed anti-MMP activity in proliferation, migration and zymography assays. Furthermore, previous studies showed that, when incorporated into a small peptide, L-DOPA exhibited zinc binding consistent with a 1:1 peptide:zinc complex^25^, and when incorporated into alcohol dehydrogenase II it facilitated an increase in Zn^2+^ binding, compared to the wild-type protein^31^. It was also shown that HqAla binds divalent ions, such as Zn^2 + 32,33^, Cu^2+ 33^ and Ca^2 + 34^, and that incorporation of HqAla into different proteins increased the metal-binding capabilities of those proteins.

In this study, we thus used genetic code expansion to incorporate the bulky, polar, metal-binding NCAAs L-DOPA and HqAla into various positions in N-TIMP2 that are located near the catalytic Zn^2+^ ion or to one of the Ca^2+^ structural ions of a bound MMP. We reasoned that incorporation of a metal-binding NCAA into N-TIMP2 could increase the ability of the mutant N-TIMP2 to chelate the Zn^2+^ or Ca^2+^ ions in the catalytic domain and thereby disrupt the catalytic activity of the MMP. Furthermore, the use of a metal-binding NCAA with bulky polar residues might be expected to endow selective affinity of the NCAA-N-TIMP2 towards some MMPs in preference to others, due to differences in the catalytic domain subsites that are crucial for substrate/inhibitor binding.

## Results

### Choosing positions in N-TIMP2 for site-specific incorporation of NCAAs.

**Figure 1:**
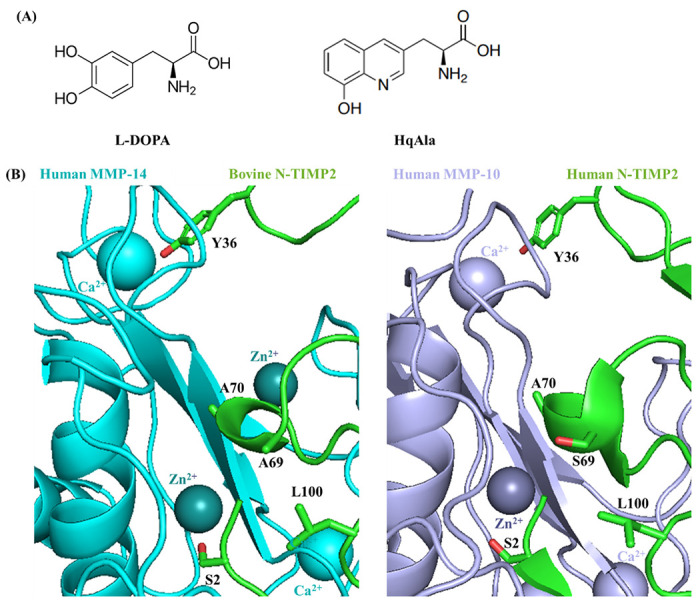
Site-directed mutagenesis of N-TIMP2 for incorporation of a NCAA. (A) Chemical structures of 3,4-dihydroxyphenylalanine (L-DOPA) and (8-hydroxyquinolin-3-yl)alanine (HqAla). (B) The available crystal structures of bovine TIMP2 (green) in complex with human MMP-14 (cyan, left) (PDB: 1BUV) and human TIMP2 (green) in complex with human MMP-10 (purple, right) (PDB: 4ILW) are used to illustrate the positions and environment of the mutated residues. The latter complex shows human N-TIMP2 with Ser in position 69. The selected mutated N-TIMP2 positions and their side chains are indicated (oxygen atoms of the side chains are colored in red).

In an attempt to enhance the specificity of N-TIMP2, we chose a strategy that rests on site-selected incorporation of a single NCAA, either L-DOPA or HqAla, at the MMP-binding interface (Fig. 1A). The thinking underlying this strategy was to exploit the steric factor that comes into play when natural amino acids are replaced with bulky NCAAs. In applying the chosen strategy, we targeted several positions in N-TIMP2 that are not only located close to the MMP’s Zn^2+^ and Ca^2+^ ions when the two proteins interact (to potentially take advantage of the metal-binding capability of the NCAA) but also interact with MMP subsites that are not highly conserved. The mutated N-TIMP2 subsites include residues S2, Y36, S69, A70 and L100 (Fig. 1B). To incorporate the NCAAs into N-TIMP2, suppression of the amber codon was performed by introducing (using PCR) a TAG codon at each of the selected positions in N-TIMP2 (one in each clone).

### Incorporation of NCAAs into N-TIMP2.

**Figure 2:**
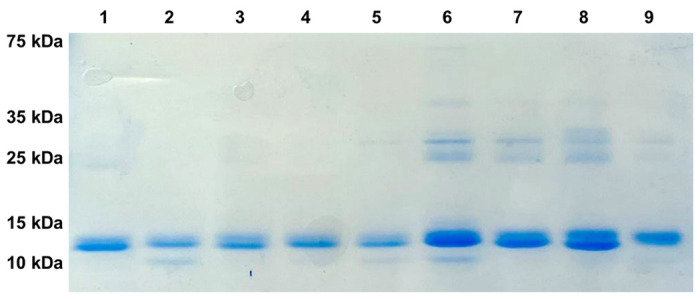
SDS-PAGE analysis of purified N-TIMP2 variants. All proteins were in the expected size of ~15 kDa. Lane 1, N-TIMP2; Lane 2, N-TIMP2-S2DOPA; Lane 3, N-TIMP2-S69DOPA; Lane 4, N-TIMP2-A70DOPA; Lane 5, N-TIMP2-L100DOPA; Lane 6, N-TIMP2-S2HqAlA; Lane 7, N-TIMP2-Y36HqAlA; Lane 8, N-TIMP2-S69HqAlA; Lane 9, N-TIMP2-A70HqAlA. Samples were run on 15% polyacrylamide gel under reducing conditions.

**Figure 3:**
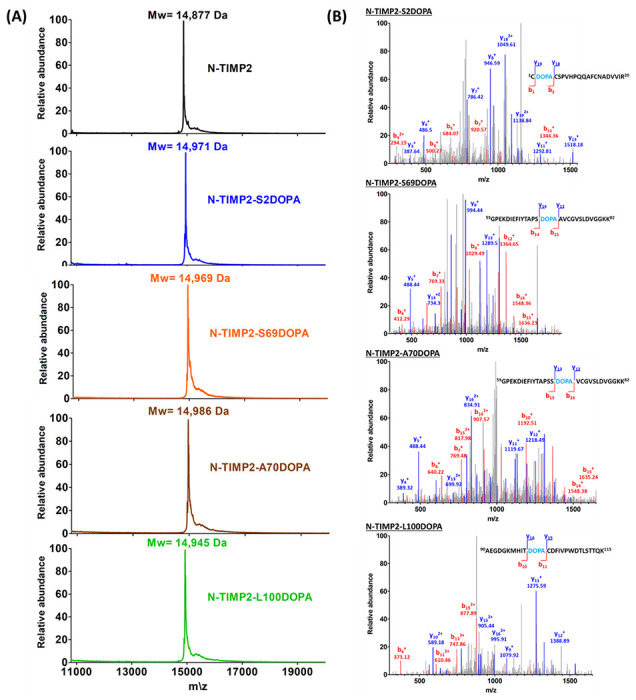
Mass spectrometry analysis of the N-TIMP2-DOPA variants. (A) MALDI-TOF analysis of N-TIMP2-S2DOPA (expected mass: 14,975 Da), N-TIMP2-S69DOPA (expected mass: 14,975 Da), N-TIMP2-A70DOPA (expected mass: 14,991 Da) and N-TIMP2-L100DOPA (expected mass: 14,945 Da), confirming the correct mass differences of ~92, ~92, ~108 and ~66 Da, respectively, between each variant and the parental N-TIMP2 (upper row, expected mass: 14,883 Da). In all cases, the differences between expected (theoretical) and observed values were less than 0.1%. (B) LC–MS/MS analysis of N-TIMP2-S2DOPA, N-TIMP2-S69DOPA, N-TIMP2-A70DOPA and N-TIMP2-L100DOPA confirming the incorporation of DOPA at a particular position in each clone.

**Figure 4:**
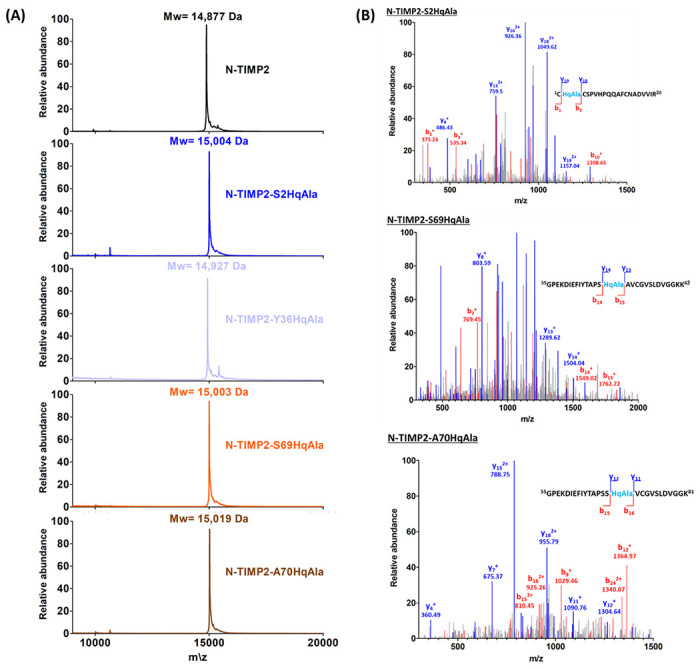
Mass spectrometry analysis of N-TIMP2-HqAla variants. (A) MALDI-TOF analysis of N-TIMP2-S2HqAla (expected mass: 15,010 Da), N-TIMP2-Y36HqAla (expected mass: 14,934 Da), N-TIMP2-S69HqAla (expectedmass: 15,010 Da) and N-TIMP2-A70HqAla (expected mass: 15,026 Da), confirming the correct mass differences of ~127, ~51, ~127 and ~143 Da, respectively, between each variant and N-TIMP2 (upper row, expected mass: 14,883 Da). In all cases, the differences between expected (theoretical) and observed values were less than 0.1%. (B) LC–MS/MS analysis of N-TIMP2-S2HqAla, N-TIMP2-S69HqAla and N-TIMP2-A70HqAla confirming the incorporation of HqAla at the particular position in each clone.

For amber suppression and incorporation of L-DOPA or HqAla into different positions in N-TIMP2, we co-transformed *Escherichia coli* strain WK6 with two plasmids, as described in the Methods section. Wild-type N-TIMP2 and the N-TIMP2-DOPA and N-TIMP2-HqAla variants were produced in the bacteria and purified using affinity chromatography (Fig. 2). Mass spectrometry confirmed the successful incorporation of L-DOPA and HqAla at each one of the selected N-TIMP2 positions. Specifically, MALDI-TOF analysis showed the expected mass difference due to each substitution, compared to N-TIMP2 (Fig. 3A and 4A). To confirm the incorporation site of the relevant NCAA in each variant, N-TIMP2-DOPA and N-TIMP2-HqAla variants were further analyzed by LC-MS/MS following trypsin digestion. The MS/MS spectrum of the peptide fragments that include the L-DOPA or HqAla incorporation site for each variant confirmed the successful incorporation of the NCAA at each of the selected positions (Fig. 3B and 4B).

### MMP inhibition by N-TIMP2-DOPA and N-TIMP2-HqAla variants.

**Figure 5:**
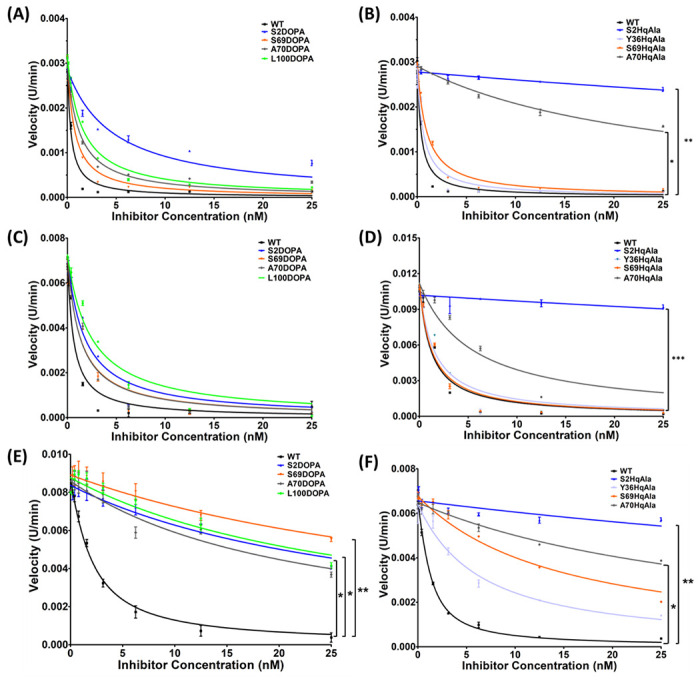
MMP inhibitory activity of N-TIMP2 variants. MMP-2_ACT_ (A and B), MMP-9_CAT_ (C and D) and MMP-14_CAT_ (E and F) were incubated with N-TIMP2, N-TIMP2-DOPA variants (A, C, E) or N-TIMP2-HqAla (B, D, F) variants at various concentrations. Cleavage of the chromogenic substrate (100 μM) was measured over time, and the velocity (slope) of the reaction as a function of inhibitor concentration was fitted to Morrison’s equation (Eq. 1) to obtain the inhibition constant *K_i_*. Statistical analysis for the comparison of N-TIMP2 to N-TIMP2-DOPA and N-TIMP2-HqAla variants was performed by Student’s t-test; *P < 0.05, **P< 0.01. Error bars represent SEM; *n* = 3.

To assess the potency of N-TIMP2-DOPA and N-TIMP2-HqAla variants in inhibiting MMP activity, an MMP activity assay was performed, in which activated MMP-2 (designated MMP-2_ACT_) and the catalytic domains of MMP-9 and MMP-14 (designated MMP-9_CAT_ and MMP-14_CAT_, respectively) were incubated with various concentrations of N-TIMP2 variants (0-25 nM) and an MMP chromogenic substrate, and the cleavage of the substrate as a function of time was measured. To determine the inhibition constants (*K_i_*), the slope of each catalytic reaction was calculated and fitted to Morrison’s tight binding equation (Fig. 5, Table 1). None of the variants containing NCAAs showed improved inhibition toward any of the MMPs tested. However, as intended, the substitutions diminished the inhibitory activity toward the different MMPs (although to widely varying extents), resulting in an enhancement of specificity. Whereas N-TIMP2 bound MMP-14_CAT_ with a *K_i_* of 0.71 nM (Table 1), a finding consistent with previous studies^35,36^, nearly all the N-TIMP2-DOPA and N-TIMP2-HqAla mutants lost their inhibitory activity towards MMP-14_CAT_ by more than one order of magnitude compared to N-TIMP2. In contrast, most of the N-TIMP2-DOPA and N-TIMP2-HqAla mutants retained their inhibition potency towards MMP-2_ACT_ and MMP-9_CAT_. Notably, N-TIMP2-Y36HqAla, N-TIMP2-S69HqAla and N-TIMP2-S69DOPA exhibited the best inhibition of MMP-2_ACT_ and MMP-9_CAT_, with only a one- to twofold diminishment of potency compared to N-TIMP2. The MMP inhibition assays suggested that the selected positions within N-TIMP2 exhibit different degrees of tolerance for mutagenesis – induced by either L-DOPA or HqAla – that differentially impact their potency toward the different MMPs.

### Incorporation of L-DOPA and HqAla Into N-TIMP2 increases its specificity towards MMP-9_CAT_ and MMP-2_CAT_.

Our MMP inhibition studies revealed different degrees of tolerance for mutations of the selected N-TIMP2 positions in terms of retention of the inhibition potency for the different MMPs. We observed that the inhibitory activity of most N-TIMP2-DOPA and N-TIMP2-HqAla variants was retained for MMP-2_ACT_ and MMP-9_CAT_, but lost for MMP-14_CAT_. To compare the degree of preference of each N-TIMP2 variant for MMP-2_ACT_ or MMP-9_CAT_ relative to MMP-14_CAT_, we calculated an inhibition specificity ratio as the ratio between the affinity (*K_i_*) of each N-TIMP2 variant for MMP-2_ACT_ or MMP-9_CAT_ divided by the *K_i_* for MMP-14_CAT_ (Table 2). All N-TIMP2 variants, except for N-TIMP2-S2HqAla and N-TIMP2-A70HqAla, showed increased specificity for both MMP-2_ACT_ and MMP-9_CAT_ vs. MMP-14_CAT_, in comparison to N-TIMP2. Notably, N-TIMP2-S69HqAla and N-TIMP2-S69DOPA showed the strongest specificity for MMP-2_ACT_, with inhibition specificity ratios of 18.96 and 46.61, respectively, and for MMP-9_CAT_, with inhibition specificity ratios of 25.59 and 45.85, respectively.

### Molecular modeling of MMPs bound to N-TIMP2-DOPA and N-TIMP2-HqAla variants.

**Figure 6:**
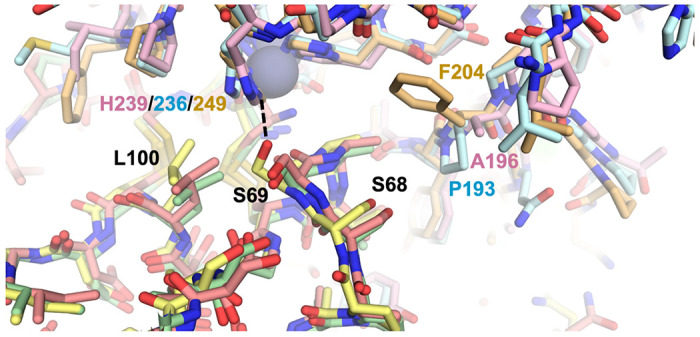
Structural comparison of MMP/N-TIMP2 complexes. Modeled complexes of N-TIMP2 with each MMP are shown, with MMP-2/N-TIMP2 in pink/salmon, MMP-9/N-TIMP2 in pale cyan/pale green, and MMP-14/N-TIMP2 in yellow orange/pale yellow. The MMP catalytic Zn^2+^ is shown as a gray sphere. The H-bond between N-TIMP2-S69 and MMP-14-H249 is shown as a dashed black line, and the relevant residues in both proteins are labeled.

To investigate the structural basis for the selectivity enhancements of N-TIMP2-S69HqAla and N-TIMP2-S69DOPA variants toward MMP-2 and MMP-9 in preference to MMP-14, we used molecular modeling approaches. The crystal structure of MMP-14_CAT_ bound to TIMP2 (1BUV)^37^ was used as a template. Superposition on the template of experimental structures for MMP-2_CAT_ and MMP-9_CAT_ showed minimal global differences in the MMP catalytic domain and facile accommodation of TIMP2 with few or no clashes. The TIMP2 chain in each complex was truncated to include only the residues of N-TIMP2, and then Ser69 was mutated *in silico* to HqAla or L-DOPA. Next, all nine model complexes (N-TIMP2, N-TIMP2-S69HqAla, or N-TIMP2-S69DOPA bound to MMP-2_CAT_, MMP-9_CAT_ or MMP-14_CAT_) were subjected to molecular dynamics-based relaxation as outlined in the Methods section. Uniquely, among the three models involving wild-type N-TIMP2, the MMP-14/N-TIMP2 model possessed a hydrogen bond between Ser69 of N-TIMP2 and His249 of the MMP (Fig. 6), suggesting that Ser69 may be a significant determinant of affinity toward MMP-14. With the exception of this specific interaction, the complexes were overall very similar. We also observed that a nearby MMP residue at the equivalent position 196/193/204 (numbering from initiator methionine for all enzymes) was not conserved, being alanine in MMP-2, proline in MMP-9, and phenylalanine in MMP-14.

**Figure 7:**
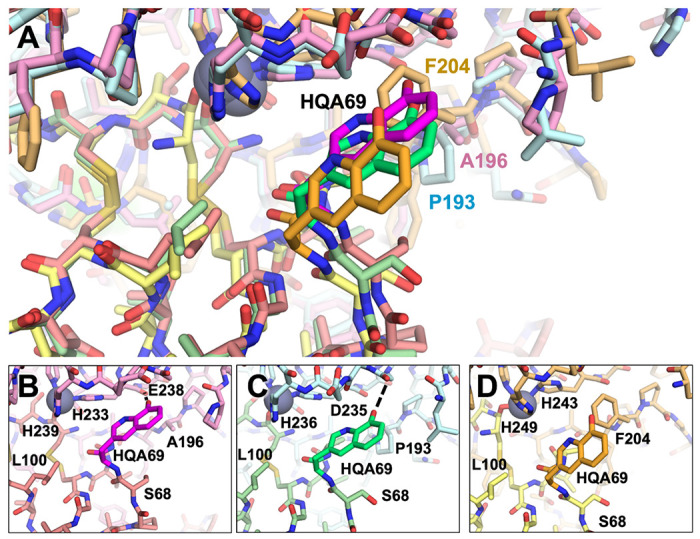
Structural comparison of MMP/N-TIMP2-S69HqAla complexes. (A) Superpositions of the modeled complexes of N-TIMP2-S69HqAla bound to each of the three MMPs are shown, with MMP-2/N-TIMP2-S69HqAla in pink/salmon, MMP-9/N-TIMP2-S69HqAla in pale cyan/pale green, and MMP-14/N-TIMP2-S69HqAla in yellow orange/pale yellow. The MMP catalytic Zn^2+^ is shown as a gray sphere. (B-D) The local environment surrounding HqAla is shown for the complex with MMP-2 (B), MMP-9 (C), or MMP-14 (D). HqAla is represented in magenta, lime, and orange, respectively, with potential H-bonds indicated with black dashed lines. HqAla appears to interact more closely with MMP-2 and MMP-9, whereas a steric clash with Phe204 precludes close interaction with MMP-14.

The modeling with the HqAla variants revealed the importance of MMP sequence differences at positions 196/193/204 (Fig. 7A). In both MMP-2- and MMP-9-bound complexes, HqAla was predicted to fit into a binding cleft adjacent to the His-liganded catalytic Zn^2+^, thereby making favorable contacts, including potential H-bonds with the Gly236/Leu237 backbone of MMP-2 (Fig. 7B) and the Gly233/Leu234 backbone of MMP-9 (Fig. 7C). In contrast, in the complex with MMP-14, the bulkier Phe204 blocked access of the NCAA to the binding cleft occupied by HqAla in the complexes with MMP-2 and MMP-9 and resulted in local shifts in the backbones of both N-TIMP2-S69HqAla and MMP-14; in this case, there were no H-bonds of HqAla with MMP-14 (Fig. 7D). Overall, HqAla could thus form strong contacts with MMP-2 and MMP-9, but only minimal contact with MMP-14.

**Figure 8:**
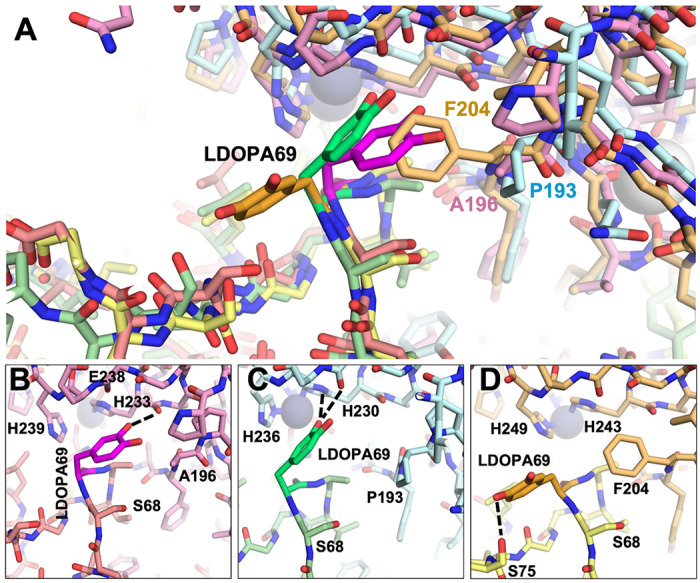
Structural comparison of MMP/N-TIMP2-S69DOPA complexes. (A) Superposition of modeled complexes of N-TIMP2-S69DOPA bound to each MMP are shown, with MMP-2/N-TIMP2-S69DOPA in pink/salmon; MMP-9/N-TIMP2-S69DOPA in pale cyan/pale green; MMP-14/N-TIMP2-S69DOPA in yellow orange/pale yellow. The MMP catalytic Zn^2+^ is shown as a gray sphere. (B–D) The local environment surrounding L-DOPA is shown for a complex with MMP-2 (B), MMP-9 (C), or MMP-14 (D). L-DOPA is represented in magenta, lime, or orange, respectively, with potential H-bonds indicated by black dashed lines. L-DOPA is predicted to interact more closely with MMP-2 and MMP-9, whereas steric clash with Phe204 favors an alternative L-DOPA rotamer that does not interact with MMP-14.

The modeling with the L-DOPA variants corroborated the impact of the MMP sequence differences at positions 196/193/204 (Fig. 8A). With MMP-2 and MMP-9, L-DOPA was predicted to fit in close to the active site adjacent to the His-liganded catalytic Zn^2+^, unobstructed by Ala196 in MMP-2 or Pro193 in MMP-9. In the complex with MMP-2, L-DOPA69 was predicted to form a potential H-bond with the backbone of MMP-2 His233 (Fig. 8B), while with MMP-9, L-DOPA69 interactions included potential H-bonds with the backbone of Leu234 and side chain of His230 (Fig. 8C). In contrast, the Phe204 of MMP-14 prevented L-DOPA69 from accessing the binding cleft, and instead the modeling protocol predicted a rotamer pointing away from MMP-14 and potentially forming an intramolecular H-bond with Ser75 of N-TIMP2-S69DOPA (Fig. 8D). Overall, substitution of L-DOPA69 conferred novel strong interactions with either MMP-2 or MMP-9 but did not stabilize the interaction with MMP-14.

## Discussion

This study presents a strategy for improving inhibitor specificity for individual enzymes within a homologous family, via mutagenesis of selected residues that participate in inhibitor-enzyme interactions. MMP family members share a common multi-domain structure, but exhibit differences in the subsites of their catalytic domains that lead to a variety of specificities for different substrates and inhibitors. As MMPs are zinc- and calcium-dependent enzymes, a potential strategy for inhibiting MMPs could be to target these cations to disrupt their coordination by MMP residues. Since MMP subsites differ in their size and shape, the size and volume of amino acids within ligands and potential inhibitors, such as TIMP family members, will affect their affinity and selectivity towards different MMPs. In the current study, two approaches were combined to manipulate the affinity and selectivity of TIMP-2 for different MMPs. In the first, the NCAAs L-DOPA and HqAla, which possess metal-binding capacity and have large side-chains, were incorporated into N-TIMP2 at selected positions with the potential to interact strongly with the Zn^2+^ and Ca^2+^ ions in the MMP catalytic domain. In the second approach, L-DOPA and HqAla were strategically placed to interact differently with distinctive subsites of different MMPs and hence to confer selectivity in binding and inhibition potencies. Incorporation of the bulky, polar, metal-binding NCAAs L-DOPA and HqAla at selected positions in N-TIMP2 did not improve its inhibition potency towards the examined MMPs–observations that highlight the important role played by the selected N-TIMP2 positions in the TIMP/MMP interactions. Nevertheless, the findings that the inhibitory activity towards MMP-14_CAT_ was significantly impaired for all N-TIMP2-DOPA and N-TIMP2-HqAla variants, but was retained for MMP-2_ACT_ and MMP-9_CAT_ for most variants, emphasize the potential of these positions to alter N-TIMP2 selectivity towards different MMPs.

An examination of the different substitution positions within N-TIMP-2 provides explanations for our findings. Position Ser2 of N-TIMP2, which is located in the N-terminal segment (residues Cys1-Pro5), is involved in the direct interaction between N-TIMP2 and the MMP catalytic pocket^13^ and is therefore is intolerant of mutation to a bulkier residue, as we observed for the substitutions with either L-DOPA or HqAla and as was previously shown for other substitutions, such as S2E^38^ and S2D^39^, at this position. Our results are thus in line with these findings, as Ser2 substitutions with both NCAAs led to significantly decreased affinities towards all tested MMPs, with a *K_i_* fold > 13, except for the retention of MMP-9_CAT_ inhibition by N-TIMP2-S2DOPA. Notably, N-TIMP2-S2HqAla showed > 2 orders of magnitude decrease in the affinity towards all three MMPs, suggesting that the bulky side chain of HqAla, compared to Ser, interferes with the MMP binding, probably due to steric hindrance.

Position Y36 of N-TIMP2 is located on the tip of the AB loop (residues D30-K41) and interacts with MMP-14 at a site that is distant from the catalytic pocket^40,41^. Different mutations (Y36F, Y36G and Y36W) led to decreased affinities towards MMP-14, exhibiting significant higher inhibition constants (*K_i_*-fold of 15 to 103) and lower association rate constants (K_on_-fold of 36 to 180), compared to N-TIMP2, whereas their affinities towards MMP-2 were maintained^42^. Our results extend these previous findings, as N-TIMP2-Y36HqAla showed a 6.64-fold decreased affinity towards MMP-14_CAT_, whereas it maintained its inhibitory potency towards both MMP-2_ACT_ and MMP-9_CAT_, compared to N-TIMP2.

Positions S69 and A70 are located on the surface-exposed C-connector loop of N-TIMP2 (residues Ser68-Cys72^41^) that interacts with the MMP catalytic domain. The crystal structures of the TIMP2/MMP-13 and TIMP2/MMP-14 complexes suggest that this loop may have different affinities towards each MMP, as MMP-14 forms favorable contacts with it and MMP-13 repulses it, via residues A66 and V71 in TIMP2 and the bulky Y176 residue in MMP-13 compared to the smaller T190 in MMP-14^43^. Our structural modeling corroborates the distinct nature of the interactions occurring between this loop and the different MMPs examined here, due, in particular, to the bulkier Phe204 residue of MMP-14, at a position where MMP-2 and MMP-9 possess the smaller residues Ala196 and Pro193, respectively. This difference appears to explain much of the decreased affinity towards MMP-14_CAT_ for both the bulky L-DOPA and HqAla substitutions at position 69, in comparison to Ser at position 69 of N-TIMP2. Previous computational analysis of binding landscapes for the interactions between N-TIMP2 with MMP-9_CAT_, in which selected positions in N-TIMP2 were randomly mutated, has shown position S69 to be tolerant to randomization^44^, which may further explain the retention of inhibition potency towards MMP-9_CAT_ upon substitution with either L-DOPA or HqAla at this position.

Position L100 on N-TIMP2 is located on the EF loop between two beta-strands, sE and sF^37^. In this loop – previously identified as one of the N-TIMP2 binding sites for MMP-3^45^ and MMP-14 – L100 is in close proximity to the MMP-14 catalytic Zn ion^44^, which may explain the observed reduction (by ~ 20-fold) in affinity towards MMP-14_CAT_ upon substitution of L100 with L-DOPA.

Our molecular modeling reveals how local sequence differences between the MMPs lead to differential susceptibility to inhibition by N-TIMP2 variants with insertion of the bulky HqAla or L-DOPA in position 69. Specifically, MMP-14 is much less susceptible to inhibition by the variants as a consequence of deleterious steric interactions between HqAla or L-DOPA and MMP-14 Phe204, a position occupied by smaller residues in MMP-2 and MMP-9. Overall, this work suggests that different TIMP/MMP complexes have differential ability to tolerate the introduction of bulky residues within interface positions. In the absence of crystal structures, molecular dynamic simulations can be used to elucidate the molecular basis for these differences in selectivity.

In summary, in this study, the properties of HqAla and L-DOPA that shaped their differential interactions with the different MMPs can be attributed to their bulkiness and ability to form polar interactions, rather than to their known metal-binding capability. In the future, however, our approach might be extended to take advantage of metal coordination by metal-binding NCAAs at the interface of N-TIMP2 with its MMP targets (perhaps by choosing other metal-binding NCAAs and other positions within N-TIMP2) in order to improve N-TIMP2 potency towards different MMPs. This approach may also be used to optimize and modulate binding interactions of other protein complexes involving other metalloproteins.

## Materials And Methods

### Generation of N-TIMP2-DOPA and N-TIMP2-HqAla variants.

To choose the positions for the incorporation of L-DOPA or HqAla within N-TIMP2, the crystal structures of TIMP-2-MMP-14 (PDB 1BUV) and TIMP-2-MMP-10 (PDB 4ILW) complexes were analyzed in PyMol (The PyMOL Molecular Graphics System, Version 1.1 Schrodinger, LLC.). The gene encoding for N-TIMP2 (positions 1-127) was cloned into a pMECS expression vector (a kind gift from Dr. Serge Muyldermans, Vrije University Brussels, Brussels, Belgium) using restriction free PCR (RF-PCR)^46^, which served as a template for introducing the TAG point mutation in the selected positions (S2, Y36, S69, A70 and L100) of N-TIMP2. All plasmid sequences were verified by Sanger sequencing (Genetics Unit, NIBN, Ben-Gurion University of the Negev, Israel).

The following primers were used in the RF-PCR to generate the gene for each clone:

**Table:** 

Position	Primers
S2	FWD: 5′-GCCGGCCATGGCCTGCTAGTGCTCCCCGGTGCACC-3′ REV: 5′-GGTGCACCGGGGAGCACTAGCAGGCCATGGCCGGC-3′
Y36	FWD: 5′-CTCTGGAAACGACATTTAGGGCAACCCTATCAAG-3′ REV: 5′-CTTGATAGGGTTGCCCTAAATGTCGTTTCCAGAG-3′
S69	FWD: 5′-GTTTATCTACACGGCCCCCTCCTAGGCAGTGTGTGGGGTC-3′ REV: 5′-GACCCCACACACTGCCTAGGAGGGGGCCGTGTAGATAAAC-3′
A70	FWD: 5′-GTTTATCTACACGGCCCCCTCCTCGTAGGTGTGTGGGGTCTC-3′ REV: 5′-GAGACCCCACACACCTACGAGGAGGGGGCCGTGTAGATAAAC-3′
L100	FWD: 5′-CAAGATGCACATCACCTAGTGTGACTTCATCGTG-3′ REV: 5′-CACGATGAAGTCACACTAGGTGATGTGCATCTTG-3′

### Production and purification of N-TIMP2-DOPA and N-TIMP2-HqAla variants.

pMECS plasmids encoding the different clones of N-TIMP2 were co-transformed into *E. coli* strain WK6, with either one of the following plasmids: (i) pAC-DHPheRS6TRN plasmid, containing the DHPheRS/Mj-tRNA_CUA_ genes for L-DOPA incorporation^47^ or (ii) pEVOL-HqAlaRS plasmid, containing the HqAlaRS/Mj-tRNA_CUA_^Tyr^ genes for HqAla incorporation^32^, both for TAG suppression. The bacteria were grown with stirring at 200 rpm at 37°C in TB medium (17 mM KH_2_PO_4_, 94 mM K_2_HPO_4_, 12 g/L peptone, 24 g/L yeast extract, 0.4% glycerol) containing: 100 μg/ml ampicillin for N-TIMP2; 100 μg/ml ampicillin, 10 μg/ml tetracycline and 5 mM L-DOPA (Sigma-Aldrich, Israel, added at OD_600_ = 0.4) for N-TIMP2-DOPA clones; or 100 μg/ml ampicillin, 50 μg/ml chloramphenicol and 3 mM HqAla (BLD Pharmatech Ltd., China) for N-TIMP2-HqAla clones. At an OD_600_ of 0.4, 0.2% arabinose (Mercury, Rosh Ha’ayin, Israel) was added to N-TIMP2-HqAla clones (for PylRS induction), and at an OD_600_ of 0.6–0.9 the expression of all proteins (N-TIMP2, N-TIMP2-DOPA and N-TIMP2-HqAla variants) was induced by addition of 1 mM IPTG (Sigma-Aldrich, Israel) to the medium and temperature adjustment to 28°C (for N-TIMP2 and N-TIMP2-HqAla) or 22°C (and under anaerobic conditions for N-TIMP2-DOPA) and overnight incubation. The cell pellet obtained by centrifugation at 4800 g for 30 min of 500 mL of bacterial cell culture (with a final OD_600_ < 20)] was subjected to osmotic shock using 9 mL of TES buffer (500 mM sucrose, 200 mM Tris-HCl, 0.5 mM EDTA, pH 8) for 2 h at 4°C and 200 rpm, followed by incubation overnight in 18 mL of TES buffer (diluted 1:4 in doubly distilled water) to yield soluble proteins (i.e., periplasmic extracts). The proteins were further purified using affinity chromatography on Ni-NTA gravitational beads (Invitrogen, CA, USA) and eluted with 0.5 M imidazole in phosphate buffered saline (PBS). The eluted fraction was dialyzed against PBS, and the size and purity of the proteins were evaluated by using SDS – PAGE gel electrophoresis and mass spectrometry (MALDI-TOF Reflex-IV, Ilse Katz Institute for Nanoscale Science and Technology, BGU, Israel). For LC-MS/MS analysis of N-TIMP2-DOPA and N-TIMP2-HqAla variants, excised SDS-PAGE gel bands were denatured, reduced, alkylated and digested by trypsin. Digested peptides were then subjected to tandem mass spectrometry analysis by the LTQ-Orbitrap XL ETD system (Ilse Katz Institute for Nanoscale Science and Technology Shared Resource Facility, BGU, Israel). Protein concentrations were determined by UV-Vis absorbance at 280 nm, using a NanoDrop Spectrophotometer (Thermo Fisher Scientific), with an extinction coefficient (ε280) of 13,500 M^− 1^·cm^− 1^ for all N-TIMP2 proteins.

### MMP inhibition studies.

The human MMP-9 catalytic domain (MMP-9_CAT_, residues 107–215, 391– 443) and the human MMP-14 catalytic domain (MMP-14_CAT_, residues 112–292) were purified as described previously^35^. The inhibition constants (*K_i_*) of N-TIMP2 proteins against pre-activated MMP-2 (MMP-2_ACT_; pre-activated in vitro using 4-aminophenylmercuric acetate (APMA), Sigma-Aldrich, Israel), MMP-9_CAT_ and MMP-14_CAT_ were determined as previously described^35^. The inhibition of the catalytic activity of MMP-2_ACT_ (0.6 nM), MMP-9_CAT_ (3 nM) and MMP-14_CAT_ (1 nM) was measured against the chromogenic MMP substrate, Ac-Pro-Leu-Gly-[2-mercapto-4-methyl-pentanoyl]-Leu-Gly-OC_2_H_5_ (ENZO Life Sciences, USA). MMPs were incubated with 0–25 nM of N-TIMP2, N-TIMP2-DOPA and N-TIMP2-HqAla variants in assay buffer (50 mM HEPES, 10 mM CaCl_2_, 0.05% Brij-35, 1 mM DTNB, pH 7.5) for 1 h at 37°C. Thereafter, the chromogenic substrate, at a final concentration of 100 μM, was added to the reaction, and the absorbance was monitored at 412 nm using a Synergy 2 plate reader (BioTek, USA) at 37°C for 30–60 min at 1-min intervals. Data analysis was performed according to the manufacturer’s instructions and fitted by multiple regressions to Morrison’s tight binding inhibition equation (Eq. 1), the classic competitive inhibition equation for tight binding, by using Prism (GraphPad Software). Mean values of *K*_*i*_ ± standard error of the mean (SEM) were obtained from three independent experiments. Statistical analysis was performed using Student’s t-test.

(Eq.1) $$\frac{V_{i}}{V_{0}}=1-\frac{\left(\left[E\right]+\left[I\right]+K_{i}^{app}\right)-\sqrt{{\left(\left[E\right]+\left[I\right]+K_{i}^{app}\right)}^{2}-4\left[E\right]\left[I\right]}}{2\left[E\right]}$$

where V_i_ - enzyme velocity in the presence of inhibitor, V_0_ - enzyme velocity in the absence of inhibitor, E - enzyme concentration, I - inhibitor concentration, S - substrate concentration, K_M_ - Michaelis-Menten constant, and $K_{i}^{\mathrm{app}}$ - the apparent inhibition constant, which is given by Eq. 2:

(Eq.2) $$K_{i}^{app}=K_{i}\left(1+\frac{\left[S\right]}{K_{M}}\right)$$

where *K_i_*-inhibition constant.

### MMP/TIMP modeling.

The models of MMP-2/N-TIMP2 and MMP-9/N-TIMP2 complexes were constructed by superposing the MMP-2 chain of 3AYU.pdb^48^ or the MMP-9 chain of 4JIJ.pdb^49^ onto the MMP-14 chain of 1BUV^37^. The C-terminal domain of TIMP-2 was deleted and modified catalytic residues were back-mutated to the wild-type sequence.

Mutations with L-DOPA or HqAla and incorporation sites were chosen using PyMOL^50^ and a database of NCAAs SwissSidechain^51^. The rotamer of the mutated sidechain was chosen so as to minimize clashes. Complexes of the wild type and the variants were then subjected to identical molecular dynamics simulation relaxation protocols using YASARA^52^, i.e., 500 ps of energy minimization with the YASARA2 forcefield under explicit solvation in a cubic simulation box extending 10 Å from the protein. The relaxation was carried out with the following parameters: temperature 298 K, solvent density 0.997 g/L, pH 7.4, timestep 2 fs, frames saved every 25 ps. The global energy of each resulting frame was plotted to ensure a plateau of convergence to verify that relaxation was complete. After relaxation, representative frames were chosen for structural comparisons.

## Figures and Tables

**Table 1.** Inhibition constants (*K_i_*,) for N-TIMP2 variants binding to the different MMPs.

N-TIMP2 variant	MMP-2_CAT_		MMP-9_CAT_		MMP-14_CAT_	
	*K_i_* ^a^	*K_i_* (fold)^b^	*K_i_* ^a^	*K_i_* (fold)^b^	*K_i_* ^a^	*K_i_* (fold)^b^
N-TIMP2	0.233 ± 0.022	1	0.322 ± 0.090	1	0.711 ± 0.069	1
N-TIMP2-S2DOPA	3.139 ± 0.455	13.48	0.623 ± 0.086	1.93	14.6 ± 2.2	20.59
N-TIMP2-S69DOPA	0.460 ± 0.047	1.98	0.468 ± 0.069	1.45	21.4 ± 3.7	30.15
N-TIMP2-A70DOPA	0.785 ± 0.081	3.37	0.452 ± 0.059	1.40	10.6 ± 1.6	14.89
N-TIMP2-L100DOPA	1.003 ± 0.091	4.31	0.849 ± 0.111	2.64	14.2 ± 2.1	19.99
N-TIMP2-S2HqAla	97.7 ± 14.5	419.45	70.4 ± 26.93	218.48	97.7 ± 24.2	137.47
N-TIMP2-Y36HqAla	0.413 ± 0.079	1.77	0.543 ± 0.095	1.69	4.72 ± 0.46	6.64
N-TIMP2-S69HqAla	0.606 ± 0.063	2.60	0.449 ± 0.065	1.40	11.5 ± 1.1	16.16
N-TIMP2-A70HqAla	16.7 ± 0.9	71.58	1.93 ± 0.32	5.99	27.23 ± 2.31	38.30

a *K_i_* values (nM) were obtained by fitting the data shown in Figure 5 to Morrison’s tight binding equation.

b *K_i_* (fold) is calculated as the ratio between the *K_i_* of N-TIMP2 variant and the *K_i_* of N-TIMP2.

**Table 2.** Inhibition specificity of N-TIMP2 variants.

N-TIMP2 variant	Inhibition specificity ratio^a^	
	MMP-2_CAT_	MMP-9_CAT_
N-TIMP2	3.05	2.21
N-TIMP2-S2DOPA	4.66	23.51
N-TIMP2-S69DOPA	46.61	45.85
N-TIMP2-A70DOPA	13.49	23.43
N-TIMP2-L100DOPA	14.17	16.73
N-TIMP2-S2HqAla	1.00	1.39
N-TIMP2-Y36HqAla	11.43	8.69
N-TIMP2-S69HqAla	18.96	25.59
N-TIMP2-A70HqAla	1.63	14.11

a Specificity is calculated as the ratio between the affinity (*K_i_*) for MMP-14_CAT_ in comparison with other MMPs [*K_i_* for MMP-14_CAT_/*K_i_* for MMP-2_ACT_ or MMP-9_CAT_].

## Data Availability

The datasets used and/or analysed during the current study available from the corresponding author on reasonable request.
